# Inversion utérine: à propos d’un cas

**DOI:** 10.11604/pamj.2018.29.99.13644

**Published:** 2018-02-05

**Authors:** Chimae Eddaoudi, Mohammed Achraf Grohs, Abdilhay Filali

**Affiliations:** 1 Maternité des Orangers, Université Mohammed V, Rabat, Maroc

**Keywords:** Inversion utérine, hémorragie de la délivrance, désinvagination, Uterine inversion, hemorrhage after delivery, disinvagination

## Abstract

Une inversion utérine est une complication rare de l’accouchement potentiellement grave, dans laquelle le corps utérin se retourne en doigt de gant et fait saillie dans le vagin ou hors de la vulve. Cette pathologie se manifeste en général juste après l’accouchement, par une douleur importante dans un tableau de choc hémorragique. Le diagnostic est essentiellement clinique et impose d’être immédiat afin de permettre une réinversion rapide avant la formation d’un anneau de striction. En effet, la mortalité est aujourd’hui faible si le diagnostic et la prise en charge sont précoces. L’inversion utérine ne semble pas non plus grever le pronostic obstétrical. Parmi les facteurs favorisants, on retrouve avant tout une hypotonie utérine associée à une insertion fundique du placenta, ce qui provoque une dépression du fond utérin en cas de manoeuvres intempestives (traction sur le cordon, expression utérine). La réinversion doit être rapide, menée de façon conjointe aux mesures de réanimation (traitement du choc). Elle fait appel à plusieurs méthodes manuelles consistant à retourner l’utérus après éventuelle utilisation de procédés myorelaxants (dérivés nitrés, bêtamimétiques, anesthésie générale). L’échec conduit à un traitement chirurgical par voie haute ou voie basse. Nous rapportons le cas d’une inversion utérine totale qui s’est produite avant la délivrance, lors d’un accouchement par voie haute.

## Abstract (en)

Uterine inversion is a rare but potentially serious complication of labour characterized by “glove-finger” introflexion of the uterine body protruding into the vagina or out of the vulva. This disease commonly occurs just after delivery and it is characterized by severe pain associated with hemorrhagic shock. The diagnosis is essentially based on clinical examination and must be immediate in order to allow quick reinversion before the formation of a constriction ring. Mortality rate is low if patients are early diagnosed and treated. Uterine inversion does not seem to affect the obstetrical prognosis. Contributing factors mainly include uterine hypotonia associated with fundal placenta causing depression of the uterine fundus in case of inappropriate maneuvers (pulling on the umbilical cord, uterine expression). Reinversion should be quick associated with resuscitation measures (shock resuscitation). It is based on several manual methods consisting of returning the uterus after possible muscle relaxants treatment (nitrated derivatives, betamimetics and general anesthesia). Failure results in surgical treatment using high or low approach. We report the case of total uterine reinversion during delivery by cesarean section.

## Introduction

L'inversion utérine se définit comme une invagination du fond utérin en «doigt de gant». Complication obstétricale exceptionnelle, qui engage le pronostic vital de la patiente [1]. C'est une grande urgence dont la morbidité et la mortalité dépendent de la précocité du traitement [2,3]. Sa fréquence rapportée dans la littérature varie de 1/2000 à 1/20 000 accouchements. Devant toute hémorragie du post-partum, le diagnostic d'inversion utérine aiguë doit être éliminé [4]. Nous rapportons une observation d'inversion utérine puerpérale aigue survenue après accouchement par voie haute.

## Patient et observation

**Figure 1:**
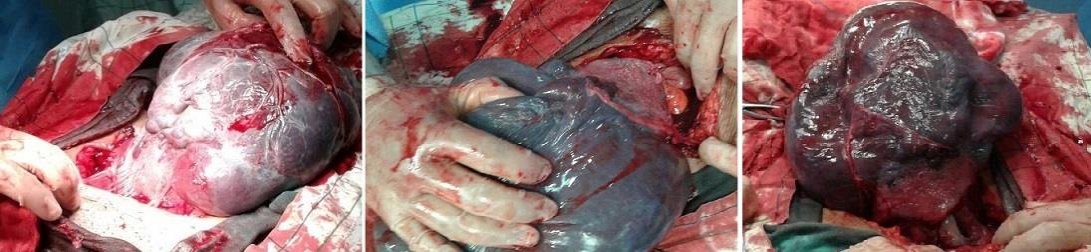
Pacenta avec utérus inversé

Mme L.I, primigeste de 22ans, sans antécédents pathologiques, grossesse bien suivie, d'évolution normale, admise au terme de 39 SA au début de travail (en phase de latence) pour prise en charge, l'examen général et obstétrical à l'admission est sans particularité, l'indication de la césarienne est posée 9 heures après son admission pour stagnation de la dilatation à 5 cm malgré une dynamique utérine correcte, la césarienne est réalisée sous anesthésie générale, avec extraction céphalique d'une fille pesant 3600g, Apgar 10 à la naissance, une inversion utérine totale s'est produite lors de la délivrance (Figure 1), associée à une hémorragie de la délivrance et un choc hypovolémique, on a procédé à un décollement progressif du placenta puis réintégration de l'utérus, on a pu récupérer un bon globe utérin après hystérorraphie, massage utérin et perfusion d'utérotoniques, la durée entre l'inversion de l'utérus et sa réduction est de 20 min, la patiente a reçu deux culots globulaires en per-opératoire, les suites post opératoires ont été simples et la NFS de contrôle objectivait un taux d'hémoglobine à 10,3g/dl.

## Discussion

L'inversion utérine d'origine obstétricale est un accident rarissime de nos jours en raison des précautions prises lors de l'extraction du placenta. Le traitement nécessite une collaboration pluridisciplinaire entre le gynéco-obstétricien, la sage-femme, l'anesthésiste et l'équipe paramédicale. Il repose sur trois points principaux: correction de l'état de choc, replacement intra-abdominal de l'utérus et correction de l'hypotonie utérine [4]. Il est décrit 4 stades anatomiques [5]: le stade I: dans lequel on trouve le fond utérin en cupule sans atteindre l'orifice cervical; le stade II: lorsque le fond utérin franchit l'orifice cervical; le stade III: dès qu'il s'extériorise hors du vagin; le stade IV: lorsque les parois vaginales participent au retournement. On parle d'inversion aiguë lorsqu'elle est découverte dans les 30 minutes. Au-delà, un œdème cervical se constitue formant un anneau gênant la réintégration du corps utérin: il s'agit alors d'une inversion sub-aiguë. Une inversion chronique étant définie par sa découverte au minimum 30 jours après l'accouchement [6]. La fréquence de l'inversion utérine est très variable selon les publications mais les plus grandes séries, provenant d'études anglo-saxonnes, estiment la fréquence de l'inversion puerpérale aiguë à 1/2 500 accouchements. Les auteurs de langue française donnent un chiffre moyen de 1/20 000 accouchements [7].

Le diagnostic de l'inversion utérine aiguë est avant tout un diagnostic clinique, effectué lors de la délivrance ou rapidement après, devant une symptomatologie d'appel rarement absente. Hémorragie, choc, douleur, réapparition d'une envie de pousser sont les signes d'appel. Un diagnostic rapide est capital car une inversion utérine engage le pronostic vital. Un traitement rapide, dès le diagnostic posé, est fondamental pour ne pas engager le pronostic vital de la patiente. Devant la possibilité de facteurs de risque iatrogènes, il est essentiel de ne jamais pratiquer certaines man'uvres visant à hâter la délivrance sur un utérus hypotonique. La réanimation médicale et la tentative de réduction manuelle (= taxis) doivent se réaliser d'emblée [7] et simultanément. La réduction manuelle de l'inversion doit être tentée le plus rapidement possible avant la formation de l'anneau cervical, qui se forme en général dans les 30 minutes après l'inversion. La manœuvre est facilitée par l'obtention d'un bon relâchement utérin obtenu par une anesthésie générale ou par l'utilisation de drogues myorelaxantes telles que les bêta-mimétiques, les dérivés nitrés ou le sulfate de magnésie. Les critères nécessaires à la constitution de l'inversion utérine, admis par tous les auteurs, sont l'hypotonie utérine et une dilatation cervicale suffisante.

Pour certains auteurs [7], 50 à 60% des inversions utérines ont comme étiologie des facteurs extrinsèques comme l'arrêt des ocytociques après un travail prolongé, la traction sur le cordon ou l'expression abdominale (manœuvre de Crédé). Des facteurs intrinsèques sont décrit comme la localisation placentaire (fundique ou accreta), la primiparité, la présence d'un cordon court, un travail long ou très rapide favorisant l'hypotonie. Cependant, il convient de citer l'étude de Watson qui ne retrouve pas de différence significative pour la parité, la longueur du travail, l'extraction instrumentale, la présentation, l'utilisation d'ocytociques pendant le travail, le délai de la délivrance, les manœuvres visant à activer la délivrance (traction sur le cordon ou manœuvre de Crédé) et le poids des enfants. Dans notre cas clinique, il s'agissait d'une patiente primigeste, ayant eu un travail de durée normale et présentant un placenta fundique normalement inséré, le facteur de risque qu'on a pu retenir est extrinsèque c'est la traction sur le cordon. Le taux de mortalité est fonction du délai de prise en charge. II varie de 80% sans traitement à une moyenne de 15% pour les inversions traitées [7]. L'hémorragie est de loin le signe le plus fréquent, elle est retrouvée dans 94% des cas dans la série de Watson [8]. Le choc est également un signe fréquent, quasi constant à partir du deuxième stade pour Thoulon, il représente environ 40% des cas, tous stades confondus [8]. Ce choc, non proportionnel à l'intensité de l'hémorragie, qu'il peut d'ailleurs précéder, s'explique d'une part par l'hypovolémie, mais aussi et surtout, par l'étirement des filets nerveux contenu dans les ligaments utérins distendus (choc neurogénique). La douleur, signe plus fréquent à partir du deuxième stade, peut être brutale et violente de siège hypogastrique. La réapparition de l'envie de pousser est un signe rare mais d'une grande valeur séméiologique car il apparaît après la délivrance [5].

Concernant notre cas la patiente était anesthésiée, le diagnostic était facile et rapide, on a assisté à une hémorragie de la délivrance associée simultanément à un état de choc corrigés par réanimation médicale (remplissage, transfusion), utérotoniques et massage utérin. Dans les inversions de stade III et IV, l'inspection périnéale retrouve une volumineuse masse rougeâtre, molle, douloureuse et sanguinolente, associée ou non au placenta. Pour notre cas, il s'agit d'une inversion survenue au cours d'un accouchement par voie haute, donc diagnostic facile et rapide, ce qui est différent des cas décrits dans la littérature, survenus après accouchement par voie basse. La réduction manuelle ou taxis consiste simplement à désinvaginer l'utérus en le repoussant progressivement. Il peut être central avec désinvagination première du fond utérin si l'anneau cervical est bien dilaté. Il sera périphérique si l'anneau cervical est plus étroit. II est indispensable de maintenir la main dans l'utérus quelques minutes pour une efficacité optimale. La méthode de Johnson qui consiste à empaumer à pleine main le fond utérin et le remonter en bloc dans la cavité abdominale jusqu'au niveau de l'ombilic, utilise la traction passive des ligaments distendus afin de désinvaginer l'utérus.Cette position est maintenue 3 à 5 minutes pour être efficace. Cette méthode, très utilisée aux États-Unis, ne compte pas d'échecs décrits [8]. La méthode hydraulique de O'Sullivan, consistant à remplir le vagin de deux litres de sérum salé chaud à 50°C pendant 5 minutes, permet la réinversion de l'utérus. Elle peut être proposée en cas d'échec de la réduction manuelle. Toutes ces méthodes de réduction se réalisent avec le placenta en place sauf si celui-ci génère une gène au repositionnement de l'utérus [8]. Après réduction manuelle, une révision endo-utérine est pratiquée et les ocytociques sont utilisés pour éviter une récidive immédiate.

Concernant notre cas, on a essayé de décoller progressivement le placenta, puis de réduire l'invagination. En cas d'échec de la réduction manuelle ou d'une récidive malgré un traitement bien conduit, une méthode chirurgicale est réalisée. L'intervention la plus fréquemment utilisée est celle décrite par Huntington. Cette technique consiste à réaliser une traction à l'aide de pinces placées les unes après les autres, sur le fond utérin, à mesure que l'on désinvagine l'utérus. Lorsque l'intervention n'est pas réalisable (anneau serré), une hystérotomie médiane postérieure est réalisée (intervention de Haultain). Par voie basse, l'intervention la plus fréquemment pratiquée est celle décrite par Spinelli. Elle consiste en une colpo-hystérotomie médiane antérieure après décollement vésico-utérin. Le choix du traitement chirurgical par laparotomie ou par voie basse se fait essentiellement en fonction de l'expérience de l'opérateur sans que l'on puisse affirmer qu'une voie est plus avantageuse que l'autre [6]. D'indication exceptionnelle, l'hystérectomie était réalisée pour un utérus gangrené sur une inversion chronique. La guérison est sans séquelles mais le risque de récidive dans les heures et jours qui suivent l'inversion peuvent s'élever à 42% lorsqu'une réduction manuelle seule a été réalisée [9]. Le risque de récurrence lors d'un accouchement ultérieur ne semble pas être augmenté, mais l'utérus gravide doit être considéré comme cicatriciel en cas de réduction chirurgicale antérieure [10].

## Conclusion

L'inversion utérine est une pathologie rare, pouvant être puerpérale ou non puerpérale. Elle est grave car elle entraine un choc hypovolémique pouvant mettre en jeu le pronostic vital. Le diagnostic et la prise en charge doivent être rapides car le pronostic en dépend. La prise en charge associe réanimation et réduction. Malgré sa rareté, tout gynécologue obstétricien doit penser à une inversion utérine devant toute hémorragie du post partum.

## Conflits d’intérêts

Les auteurs ne déclarent aucun conflit d'intérêts.
